# Early and late pulmonary effects of nebulized LPS in mice: An acute lung injury model

**DOI:** 10.1371/journal.pone.0185474

**Published:** 2017-09-27

**Authors:** Natália de Souza Xavier Costa, Gabriel Ribeiro Júnior, Adair Aparecida dos Santos Alemany, Luciano Belotti, Douglas Hidalgo Zati, Marcela Frota Cavalcante, Mariana Matera Veras, Susan Ribeiro, Esper Georges Kallás, Paulo Hilário Nascimento Saldiva, Marisa Dolhnikoff, Luiz Fernando Ferraz da Silva

**Affiliations:** 1 Laboratory of Experimental Air Pollution (LIM05), University of Sao Paulo—School of Medicine, São Paulo, São Paulo, Brazil; 2 Biochemistry Laboratory, University of Sao Paulo–School of Pharmaceutical Sciences, São Paulo, São Paulo, Brazil; 3 Laboratory of Clinical Immunology and Allergy (LIM60), University of Sao Paulo—School of Medicine, São Paulo, São Paulo, Brazil; 4 Department of Pathology, Case Western Reserve University, Cleveland, Ohio, United States of America; Hospital for Sick Children, CANADA

## Abstract

**Background and objective:**

Acute respiratory distress syndrome (ARDS) has a high mortality rate of 35–46% depending on its severity. Animal models are crucial to better understand the pathophysiology of diseases, including ARDS. This study presents a feasible animal model of acute lung injury (ALI) using nebulized lipopolysaccharide (LPS) in a non-invasive approach, focusing on its short and long-term effects.

**Methods:**

Mice received nebulized LPS or vehicle only (control group). Blood, BALF and lung tissue were collected 24 hours (LPS 24h) or 5 weeks (LPS 5w) after the nebulized LPS-induced lung injury. Inflammatory cytokines were assessed in the blood serum, BALF and lung tissue. Stereological analyses and remodeling changes were assessed by histology and immunohistochemistry at the specified time points.

**Results:**

The LPS 24h group showed increased pro-inflammatory cytokine levels, intense cell influx, increased total septal volume, septal thickening and decreased surface density of the alveolar septa. The LPS 5w group showed persistent lung inflammation, septal thickening, increased total lung volume, accentuated collagen deposition, especially of collagen type I, and decreased MMP-2 protein expression.

**Conclusion:**

We present a feasible, reproducible and non-invasive nebulized-LPS animal model that allows the assessment of both the acute and late phases of acute lung injury. The presence of lung remodeling with collagen deposition after 5 weeks makes it useful to study the pathophysiology, complications, and possible therapeutic intervention studies that aim to understand and reduce pulmonary fibrosis in the late phases of ALI.

## Introduction

Acute lung injury (ALI)/acute respiratory distress syndrome (ARDS) is a complex disease characterized by acute onset, bilateral lung infiltration, diffuse alveolar damage and protein-rich edema that can lead to severe hypoxemia and decreased lung compliance [1,2]. The most recent definition published by Ranieri et al. [2] classifies ARDS into three subgroups according to hypoxemia severity: mild, moderate and severe.

The recent Large Observational Study to Understand the Global Impact of Severe Acute Respiratory Failure reported that ARDS is responsible for 10.4% of all ICU admissions, and the mortality rate can vary from 34.9% to 46.1% depending on severity [3]. Furthermore, the exact cause of death in these patients is not easily determined, since only a small fraction of the patients die of hypoxemia [4].

To better understand the several aspects underlying ALI/ARDS, many animal models have been developed, but none can reproduce all the characteristics of the syndrome in humans. Most of these models are based on clinical conditions that are associated with the syndrome, such as sepsis, aspiration of gastric content and mechanical ventilation [5]. Therefore, different animal species, substances and methodologies have been proposed over time to mimic these conditions. Most experimental studies target only the acute phase, which is characterized by diffuse damage to the alveolar epithelium and capillary endothelium, inflammatory cell influx, protein-rich hyaline membrane formation, alveolar edema and hemorrhage [6–8]. Nevertheless, a long-term evaluation of such models is rare in the literature.

One of the widely applied models is acute lung injury induced by lipopolysaccharide (LPS). The damage caused by LPS is characterized by an acute phase, including polymorphonuclear influx, high levels of myeloperoxidase (MPO) and pro-inflammatory cytokines in the bronchoalveolar lavage fluid (BALF), and a late phase characterized by the reestablishment of cytokine levels and an increase in macrophages and lymphocytes in the BALF. Substantial hypoxia, which is a prerequisite for the diagnosis, is a parameter that is not consistent within published LPS-induced ALI models [5,9].

Most animal models using LPS are established using intratracheal instillation. The downsides of this method include the use of an invasive procedure, which is an unnatural form of contact with LPS and may cause stress to the animals and possible interference (e.g., local inflammation), as well as complications due to the surgical procedure.

Roos et al. [10] recently described a model of aerosolized LPS model and found increased neutrophilia in BALF and lung tissue and neutrophil chemoattractants chemokine (CXCL1 and 2) levels in BALB/c and C57BL/6 mice. Other authors also successfully showed that the nebulized/aerosolized LPS can reproduce the acute features of ALI/ARDS [11–13], but the late phase features are rarely studied.

There are few papers showing the long-term pulmonary effects induced by LPS administration [14,15], but most of them focus on the development of chronic diseases, thus the LPS is administered in several doses and time frames. Nevertheless, there are no papers in the literature showing significant long-term lung alterations in single dose or acute LPS induced ALI models.

In this study, we present for the first time the long-term pulmonary effects of single dose nebulized LPS ALI model, including blood and BALF cell count, gene and protein expression analysis of interleukins, and stereological analysis of the alveolar tissue structure and lung tissue remodeling. We also included the short-term effects in this model to compare with the other similar results in the literature.

## Materials and methods

This study had all ethic aspects approved by Ethics Committee on Animal Use of the University Of São Paulo—School of Medicine review board (protocol n° 177/10), including the best practices on animal manipulation and euthanasia. The animals were obtained from our University’s Animal Facility and euthanized by intraperitoneal injection of sodium thiopental (200 mg.kg-1 body weight). All mice used in this research were treated humanely according to institutional guidelines for Animal Welfare, with due consideration to the alleviation of distress and discomfort.

### Animals

Forty-eight BALB/c male mice (9 weeks old) were maintained in the animal facility at 22–26°C, 55–75% humidity and a 12/12 hour dark/light cycle with food and water *ad libitum*.

### Study design

Animals were “whole body” exposed to nebulized LPS or vehicle only (saline). During the nebulization, the animals were placed in a polysulfone box with a filter on the top, which was linked to a 100 cm long nontoxic corrugated PVC tube at one end. The other end was attached to the nebulizer (Pulmosonic Star—Soniclear^®^). To avoid contamination of the control group, we used two identical nebulization systems (S1 Fig). The final dose determination was reached after a pilot study test that included several different doses, and the final nebulization protocol consisted of a total 5 ml volume of LPS solution at 3 mg/ml concentration (Lipopolysaccharides from *Escherichia coli* 0111:B4 –Sigma-Aldrich) with sufficient time for complete solution nebulization (mean of 30 minutes). For the control group, we used 5 ml of saline solution, 0.9% NaCl. Three study groups (n = 16 each) were designed as follows:

- Control–Mice exposed to nebulized saline and euthanized after 24 hours;
- LPS 24h (acute phase)–Mice exposed to nebulized LPS (dose: 3 mg/ml; 5 ml final volume) and euthanized after 24 hours (short-term effects);
- LPS 5w (late phase)–Mice exposed to nebulized LPS (dose: 3 mg/ml; 5 ml final volume) and euthanized after 5 weeks (long-term effects).

Blood samples were collected from all animals, and lung samples from half of the animals (n = 8 per group) were frozen. From the remaining animals (n = 8 per group), BALF was collected, and the lungs were fixed in 4% buffered paraformaldehyde solution.

### Total blood, serum and BALF analysis

Total blood and BALF were subjected to full and differential cell counts. Differential cell counts were performed using May-Grünwald-Giemsa stain (300 cells per animal). The inflammatory cytokines IL-1β, IL-6, IL-10, KC and total TNF were quantified in blood serum and BALF by the cytometric bead assay (BD Bioscience, CA, USA) according to the manufacturer’s instructions. In this assay, 1200 events were acquired by a BD FACSCanto™ II flow cytometer (BD Biosciences, CA, USA) and the data analyzed in the FCAP Array software (BD Biosciences, CA, USA).

### Descriptive analysis

Slides stained with the regular Hematoxilin & Eosin (H&E) were evaluated by an experienced pathologist in order to describe the histopathological characteristics. We also did perform semiquantitative analysis of the inflammatory process using the following graduation: grade 0 (absent), 1 (discrete), 2 (mild), 3 (moderate) and 4 (intense).

### Stereological analysis

Lungs were sampled using the standardized stereological approach, fixed in formalin and embedded in paraffin. Five-micrometer thick sections were stained with H&E for lung structure analysis. The total lung volume, volume density and total volume of the lung compartments (septa, alveolar spaces and airways), the density surface and total surface area of the alveolar septa and the arithmetic mean thickness of the alveolar septum (AMT) were assessed as described in Hsia et al. [16] using the newCAST™ software (Visiopharm, Denmark).

### Molecular analysis

IL-1β, IL-6, IL-10, TNF-α, Foxp3 and MyD-88 mRNA were quantified by real-time PCR. The total RNA of the frozen lung tissue was extracted in TRIzol^®^ (Ambion^®^—Life Technologies) according to the manufacturer’s instructions. The cDNA synthesis (SuperScript^®^ VILO™ cDNA Synthesis Kit–Invitrogen—Life Technologies) and real-time PCR (Fast SYBR^®^ Green Master Mix—Applied Biosystems®—Life Technologies) were also conducted following the manufacturer’s protocol. The relative quantification of the transcripts was calculated after normalization against the levels of the reference gene RPL13a [17]. Primer sequences are presented in S1 Table.

### Immunohistochemical analysis

Tissue sections were immunostained using anti–MPO, anti-CD3 and anti-MAC2 antibodies. We counted the immunostained cells in 20 high-power fields (HPF) and calculated the proportion of cells per area of lung tissue. Lung tissue sections were also immunostained using anti-IL-1ß, anti-IL-6, anti-IL-10, anti-TNF-α, anti-collagen type I and anti-MMP2 antibodies. The inflammatory cytokines, metalloproteinases and collagen content in the lung parenchyma were quantified by measuring the proportional area (stained area/lung tissue area) in 20 HPF per animal using the software Image-Pro® Plus 4.1 (Media Cybernetics, Silver Spring, Md., USA). Immunohistochemistry antibody dilutions and commercial sources are presented in S2 Table.

### Assessment of total collagen and elastic fibers of the lung tissue

The lung tissues were stained with *Sirius Red* [18] and Weigert’s resorcin-fuchsin with oxidation [19] to assess the total content of collagen and elastic fibers, respectively. The proportional areas of collagen and elastin were quantified using the same software and strategy used for cytokines in immunohistochemistry.

### Statistical analysis

The statistical analyses were performed using the SPSS 17 software (SPSS Inc.^©^/IBM^©^ Chicago, USA). The mean, median, standard error, standard deviation and interquartile range were calculated for each variable and group. ANOVA or Kruskal-Wallis testing was performed to compare the groups according to distribution, as previously assessed by the Kolmogorov-Smirnov normality test. Tukey or Bonferroni post-hoc tests were performed also according to the data distribution. Statistical difference was assumed at the 5% significance level.

## Results

### Body weight

We observed a significant weight loss in the LPS 24h group compared to the control group (p = 0.003). The animals lost an average of 11% of body weight 24 hours after LPS administration. However, the weight was recovered after 5 weeks, with no difference between the control and LPS 5w group.

### Histopathological characteristics

**Fig 1:**
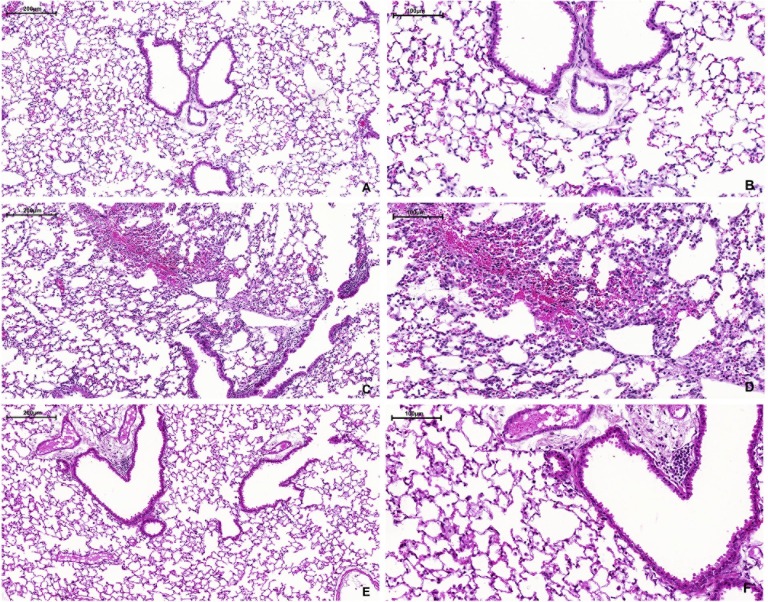
Representative photomicrographs of lung tissue (H & E staining). (A and B) Control group (10x and 20x, respectively): thin alveolar septa and no significant inflammation; (C and D) LPS 24h group (10x and 20x, respectively): intense presence of polymorphonuclear cells, alveolar thickening and focal areas of hemorrhage; (E and F) LPS 5w group (10x and 20x, respectively): alveolar thickening, persistent inflammatory cells and irregular alveolar enlargement.

Macroscopically, the lungs of all groups presented a normal aspect at the time of euthanasia. Microscopically, the lungs of the control group animals presented a healthy aspect with no signs of inflammation (semiquantitative score 0.9±0.6) (Fig 1A and 1B). In the LPS 24h group, the lung tissue showed intense perivascular, peribronchial and septal inflammatory infiltration, with a predominance of polymorphonuclear cells (semiquantitative score 3.1±0.6, p<0.05 compared to control and 5 weeks) (Fig 1C and 1D). Septal thickening, irregular distribution of air spaces and focal areas of alveolar hemorrhage were also observed. Five weeks after nebulization, the lung tissue still showed perivascular and peribronchial inflammatory infiltration with mild inflammation in the alveolar septa (semiquantitative score 1.9±0.5, p<0.05 compared to control) (Fig 1E and 1F).

### BALF and white blood cell count

**Fig 2:**
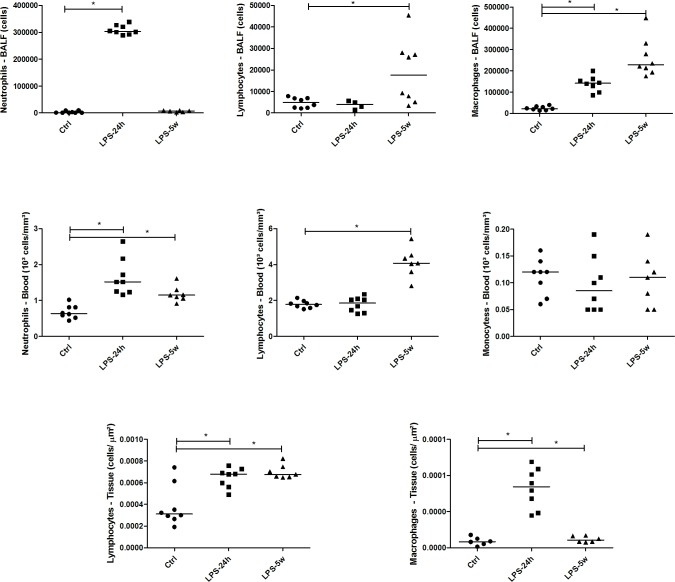
Graphical representation of total cells and differential counts present in peripheral blood, BALF and lung tissue. * p<0.05.

The white blood cell count during the acute phase (LPS 24h) showed leukocytosis (p = 0.024) predominantly due to increased neutrophils (p = 0.001) compared to the controls. The hematocrit level of the LPS 24h group was also elevated (p = 0.014). The late phase (LPS 5w) also presented leukocytosis (p = 0.0001) with predominance of both neutrophils (p = 0.0001) and lymphocytes (p = 0.0001) compared to the controls (Fig 2).

The evaluation of inflammatory cell recruitment into the bronchoalveolar space showed increased total cell counts in both LPS 24h and LPS 5w compared to the controls (p = 0.0001 and p = 0.002, respectively). The acute phase showed higher numbers of neutrophils (p = 0.0001) and macrophages (p = 0.0001) than the controls. Furthermore, the late phase showed a sustained inflammatory response with higher numbers of macrophages (p = 0.003) and lymphocytes (p = 0.031) than the controls (Fig 2).

### Inflammatory parameters

**Fig 3:**
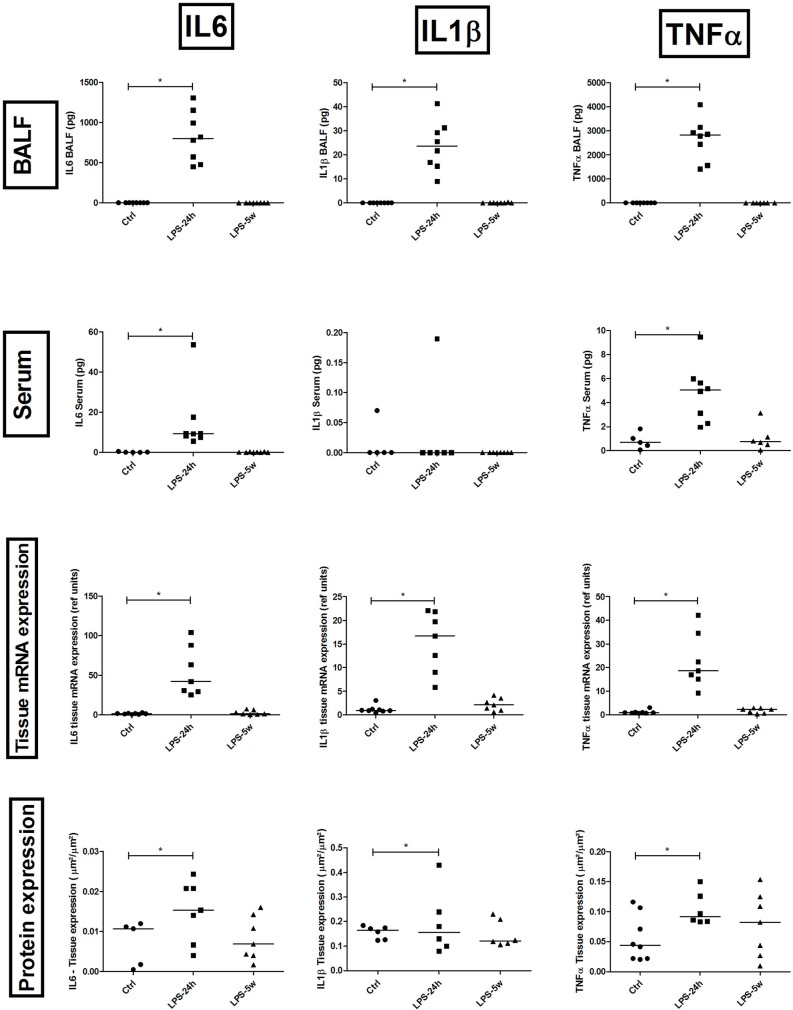
Graphical representation of quantitative results of IL-1beta, IL6 and TNF-alpha in the blood serum, BALF, protein and gene expression in lung tissue. * p<0.05.

The acute phase (LPS 24h) was characterized by a massive increase in IL-1β (p = 0.0001), IL-6 (p = 0.0001), KC (p = 0.0001) and total TNF (p = 0.0001) in the BALF as well as increased IL-6 (p = 0.002) and total TNF (p = 0.004) in the serum compared to the controls (Fig 3). The evaluation of these parameters in the lung tissue revealed similar alterations, with increased mRNA expression of IL-1β (p = 0.0001), IL-6 (p = 0.004), IL-10 (p = 0.0001) and TNF-α (p = 0.0001) and increased protein expression of IL-1β (p = 0.0001), IL-6 (p = 0.04), IL-10 (p = 0.04) and TNF- α (p = 0.022) compared to the lung parenchyma of the controls (Fig 3). Immunostained MPO positive neutrophils were significantly increased in the LPS 24h group compared to the controls (p = 0.0001) and LPS 5w (p = 0.0001). Immunostained MAC-2 positive macrophages were significantly higher in the LPS 24h group compared to the control (p = 0.007) and LPS 5w (p = 0.0001) groups (Fig 2). Additionally, CD3-positive immunostained T lymphocytes were significantly increased in the LPS 24h group compared to the controls (p = 0.0001) (Fig 2). The mRNA expression of Foxp3 was increased in the LPS 24h group compared to the control (p = 0.003) and LPS 5w groups (p = 0.04).

In the late phase, the levels of inflammatory cytokines were already normalized with no significant differences observed. In contrast, the numbers of inflammatory cells such as macrophages (p = 0.0001) and lymphocytes (p = 0.0001) were increased in the LPS 5w group compared to the controls (Fig 2). The mRNA expression of MyD-88 was increased in the LPS 24h group (p = 0.001) but not in the LPS 5w group compared to the controls.

### Stereological analysis

**Fig 4:**
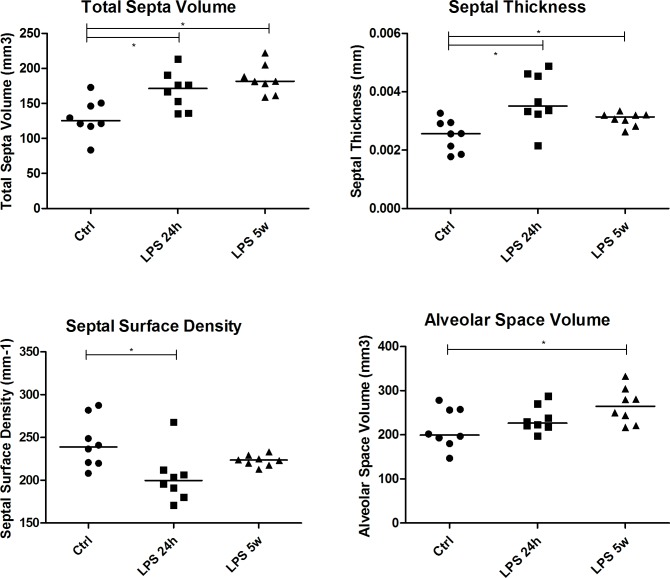
Graphical representation of total septal volume, septal thickness, septal surface density and alveolar space volume. * p<0.05.

In the acute phase (LPS 24h) there was no alteration in the total lung volume. The volume density (p = 0.015), total volume (p = 0.012) of the alveolar septa and septal thickness (p = 0.006) were increased, while the surface density of the alveolar septa was decreased (p = 0.017) compared to the control group (Fig 4). The volume density (p = 0.002) and total volume (p = 0.001) of the airway lumen were also decreased in LPS 24h compared to the controls. The volume density of the blood vessels (p = 0.027) was decreased, and there was a tendency towards a decrease in total volume (p = 0.05).

In the late phase, the total lung volume was increased in the LPS 5w group compared to the controls (p = 0.011). Some of the short-term effects were persistent even after 5 weeks of LPS-induced acute lung injury, such as increased volume density (p = 0.027) and total septal volume (p = 0.001), thickened alveolar septa (p = 0.023) and increased alveolar air space (p = 0.029) (Fig 4). We also observed decreased volume density (p = 0.002) and total volume (p = 0.021) of the airway lumen as well as increased volume density (p = 0.013) and total volume (p = 0.0001) of blood vessels in LPS 5w compared to the controls.

### Tissue remodeling

**Fig 5:**
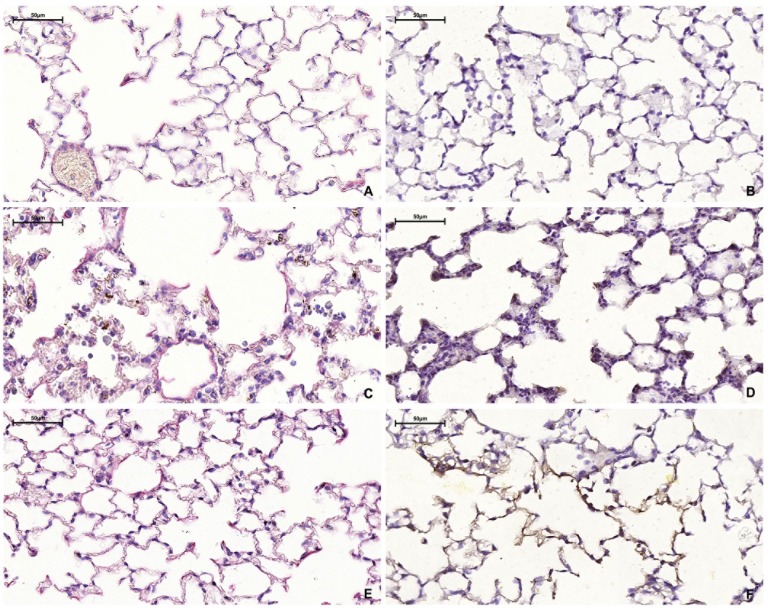
Representative photomicrographs of collagen in the lung tissue. A and B–Control group 40x (*Sirius Red* staining and immunostained collagen type I, respectively); C and D–LPS 24h group 40x (*Sirius Red* staining and immunostained collagen type I, respectively); E and F–LPS 5w group 40x (*Sirius Red* staining and immunostained collagen type I, respectively).

**Fig 6:**
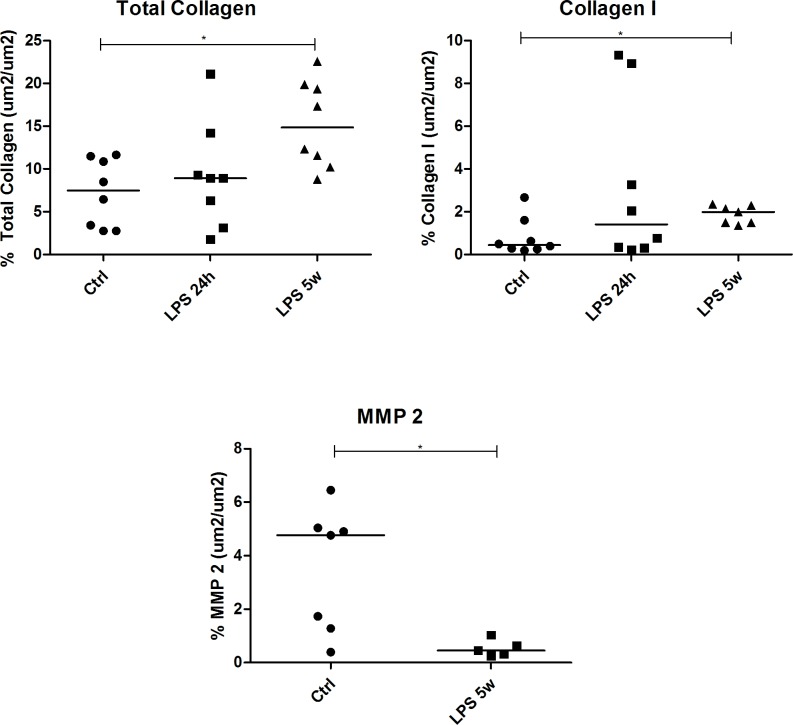
Graphical representation of lung parenchyma total collagen, collagen type I and protein expression of MMP-2. * p<0.05.

The elastic fiber content was not altered in any of the ALI phases compared to the controls. The total collagen content (p = 0.0001) and collagen type 1 proportion (p = 0.013) were increased in the lung parenchyma of the LPS 5w group compared to the controls (Figs 5 and 6). Considering these results, we also analyzed metalloproteinase (MMP) -2 by immunohistochemistry in the LPS 5w and control groups. MMP-2 expression was decreased in the LPS 5w group (p = 0.015) compared to the controls (Fig 6).

## Discussion

Human studies have offered valuable clues to understanding the pathophysiology of ALI/ARDS; however, it is difficult to control all the interfering variables and confounding factors, especially in these critically ill patients. Animal models, on the other hand, offer controlled conditions to test and validate hypotheses and mechanisms regarding the disease. LPS-induced ALI/ARDS is one of the most applicable, reliable and reproducible *in vivo* models for the study of the molecular mechanisms and potential therapies for inflammation-associated lung injury [20].

The acute phase of the ALI model described in our study shows compatible characteristics with other models previously described in the literature, despite the differences in the methodologies employed. The review presented by Matute-Bello et al. [9] presents the most important and relevant features to be considered in an ALI model, such as histological evidence of tissue injury, disruption of the alveolar-capillary barrier, and inflammatory response. In our model, these features were well characterized, exhibiting intense accumulation of inflammatory cells (especially neutrophils) in the alveolar and interstitial space; increased inflammatory cytokines in the BALF (IL-1β, IL-6, KC and TNF), tissue (IL-1β, IL-6, IL-10 and TNF) and blood serum (IL-6 and TNF); increased septal volume; alveolar septal thickening; decreased airway lumen volume; and alveolar hemorrhage.

Another review by Mühlfeld and Ochs [21] highlights the importance of edema assessment in an ALI model. Although no direct assessment of edema was performed in this study, some of the alterations could be a consequence of septal edema, such as the observed increase in septal volume and septal thickening in the LPS 24h group.

One of the most remarkable characteristics of ARDS/ALI is the parenchymal injury. An overwhelming inflammatory process in the lungs causes alveolar epithelial and endothelial injury. The epithelial injury, possibly caused by the intense influx of neutrophils through the epithelial barrier to the alveoli lumen, exposes the basement membrane and leaves it more vulnerable to bacterial action. The endothelial damage occurs as a consequence of several mechanisms, including the most recognized pathway of neutrophil-dependent injury. Neutrophils accumulate in the lung vasculature and become activated, releasing countless active substances including inflammatory cytokines. Another mechanism of endothelial injury is the action of the platelets and their interactions with neutrophils and monocytes [1, 22]. Our model exhibited signs of significant injury in the lung parenchyma with increased numbers of polymorphonuclear inflammatory cells, focal areas of hemorrhage, septal thickening and destruction. In addition, the significant rise in the expression of pro-inflammatory cytokines in the lung parenchyma, but not in the airways, suggests that the LPS injury in this model leads to diffuse alveolar damage, probably related to the massive neutrophil influx.

We also observed an increase in MPO-positive neutrophils 24 hours after LPS administration. The neutrophil MPO can activate and stimulate macrophages, worsening the inflammatory response and cytotoxic state, contributing to capillary and alveolar damage [23].

Most LPS-induced acute lung injury models show a recovery of the inflammatory parameters to the basal levels at 48 hours after the LPS administration [5], whereas in our model, some of these parameters persisted for longer periods. Furthermore, most studies evaluate the LPS response no longer than 24 hours after the LPS administration. Yoshida et al. [24] showed that the total cell count has its peak at approximately the 5^th^ day after LPS administration and returns to the basal level by the 14^th^ day. Our study showed that 5 weeks after the LPS administration, the BALF total cell count was still altered in comparison with the controls, and the blood leukocytes and lymphocytes were also still increased. The absence of neutrophils, reduced IL-1β, IL-6 and KC and the presence of lymphocytes and macrophages in the BALF suggest the maintenance of the inflammatory response with a more chronic cellular profile.

The role of Toll-like receptor 4 and MyD-88 in the LPS-induced inflammatory response is well described in the literature [25–28]. Our study also showed that the mRNA expression of MyD-88 was increased in the LPS 24h group but not in the LPS 5w group compared to the controls, suggesting that the acute inflammatory stimuli of the LPS have subsided.

Inflammation resolution is a complex and active process that does not merely involve the catabolism of inflammatory mediators and abrogation of inflammatory processes [29]. According to the study by D’Alessio et al. [30], lymphocyte-deficient Rag-1^-/-^ and wild-type mice exhibit similar LPS-induced injury extension. Nonetheless, the Rag^-/-^ mice showed an impairment of lesion recovery, suggesting that lymphocytes did not determine the initial injury severity, but the injury resolution required them. In addition, the regulatory lymphocyte (Treg) subpopulation promotes inflammation resolution via pro-repair effects on macrophage inflammatory function, neutrophil efferocytosis, epithelial proliferation and the limitation of fibroproliferation [30–32]. Our model showed increased mRNA Foxp3 expression in the lung tissue of the LPS 24h group. Although the Foxp3 expression found in this study may not exclusively represent T-reg cells, there are evidence that mRNA, protein and immunostained Foxp3 expression are correlated [33]. This might, at least partially, suggest that this counter regulatory process could begin during the acute inflammation.

Animal models of ALI/ARDS should reproduce the mechanisms and consequences of injury in humans, including physiological and pathological changes that occur over time. Due to changes in the pulmonary response and morphology over time, the evolution of the lesion and its repair should also be present in the model [5]. In addition to the persistent inflammatory response observed 5 weeks after the LPS administration, the most remarkable feature of the LPS 5w group was the tissue remodeling with collagen deposition, a feature rarely reported in LPS-based animal models of ALI. It is interesting to note that most of the LPS induced ALI models only evaluate the acute outcomes so most of the times the 5-week period is not even evaluated. Although some authors report that the lung injury resolves in less than 14 days [24] they usually only refer to inflammatory aspects, not tissue remodeling.

In humans, the rate of ARDS recovery and the underlying conditions that could lead to healing with lung fibrosis are highly variable and controversial [34]. Collagen deposition should not be interpreted solely as a late tissue response to abnormal scarring, since ARDS patients show increased procollagen levels during the first 48 hours after diagnosis [35, 36]. Lung fibrosis is more frequently observed in patients who survive two weeks or more, and the lung collagen content more than doubles during this period [37, 38]. Moreover, pro-inflammatory cytokines, such as TNF-α and IL-1β, secreted during the acute phase are potentially fibrogenic, and the substitution of collagen type III with collagen type I occurs during the course of ARDS [37]. The LPS 5w group nearly doubled the collagen content in the lung parenchyma, and low MMP-2 levels may have reduced denatured collagen and collagen fragments degradation [39]. We also observed a higher content of type 1 collagen in the late phase of our model. All these features are consistent with the ARDS pathophysiology described in humans. These findings become even more interesting if we take into account the fact that the BALB/c mice are generally described as a strain resistant to pulmonary fibrosis [40].

Bleomycin is one of the agents most widely used to model lung fibrosis (mimicking chronic pulmonary fibrosis) [41]. Some papers reported its potential use in ALI in order to reproduce the late phase effects [42]; however, it may be questioned based on claims that it is an overwhelming stimulus and has little relevance to the clinical scenario [5]. In addition, bleomycin may require repeated administration to achieve the desired outcome [41].

A less invasive LPS administration route minimizes animal discomfort and stress as well as any alterations/complications due to the surgical procedure. Any surgery can cause a secondary systemic and local inflammatory response, thus requiring the use of drugs to control the pain to prevent unnecessary suffering of the animals. In addition, many analgesics used for postoperative pain control also have an anti-inflammatory effect. According to our dose tests conducted in a pilot study, another observed advantage of the nebulized LPS was that the lesion was evenly distributed throughout the lung tissue compared to intratracheal instillation, decreasing the probability of LPS accumulation in the airways. Keeping the respiratory rate nearly normal may also be a factor that influences the injury distribution, since there is no need to anesthetize the animals for this procedure.

ARDS/ ALI is a disease that predominantly affects the lung parenchyma. Our data demonstrated that our model was able to reproduce this feature and that the inflammatory response is mostly located in the lung parenchyma and not in the airways. We considered this model very feasible and reproducible with a relatively low cost (using a custom-built exposure chamber and a common nebulizer). However, during our dose tests, one limitation observed was that for doses higher than 5 mg/ml of LPS, the solution becomes harder to nebulize using the common nebulizer.

## Conclusion

In conclusion, the nebulized LPS ALI model is a feasible, reproducible and non-invasive animal model that allows the assessment of the acute and late phases of acute lung injury.

The presence of lung remodeling with collagen deposition after 5 weeks makes it useful to study the pathophysiology, complications, and possible therapeutic intervention studies that aim to understand and reduce pulmonary fibrosis in the late phases of ALI.

## Supporting information

S1 Fig Exposure system consisting of nebulizer and exposure chamber. (TIF)

S1 Table Primer sequences. (DOCX)

S2 Table Immunohistochemistry standards. (DOCX)
