# Supplementary figures and images for: Early and late pulmonary effects of nebulized LPS in mice: An acute lung injury model
Source: PLoS One. 2017 Sep 27;12(9):e0185474. doi: 10.1371/journal.pone.0185474 (PMC5617199; doi:10.1371/journal.pone.0185474)

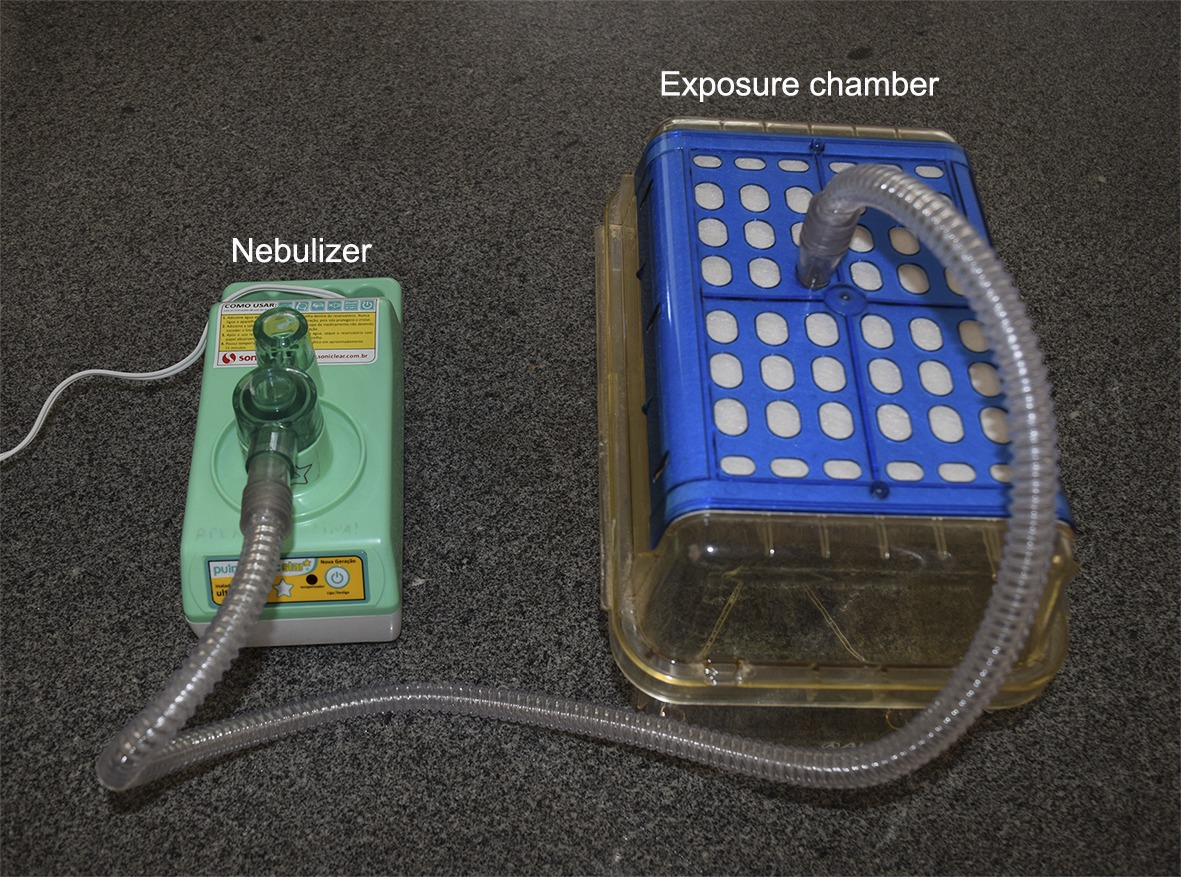

Supplement: S1 Fig — (TIF) [file pone.0185474.s001.tif]
