# Supplementary material for: Early and late pulmonary effects of nebulized LPS in mice: An acute lung injury model
Source: PLoS One. 2017 Sep 27;12(9):e0185474. doi: 10.1371/journal.pone.0185474 (PMC5617199; doi:10.1371/journal.pone.0185474)
Supplement: S1 Table — (DOCX) [file pone.0185474.s002.docx]

**S1 Table. Primer sequences**

| **Gene** | **Forward Sequence** | **Reverse Sequence** |
| --- | --- | --- |
| **RPL13a** | AACCTTTGGTCCCCACTTCCCT | TCCTCAAGACCAACGGACTCCT |
| **IL-1β** | TTCCCATTAGACAACTGCACTAC | GTCGTTGCTTGGTTCTCCTT |
| **IL-6** | TGTGCAATGGCAATTCTGAT | TCTGCTGTCTTTGGGACCTTGTC |
| **IL-10** | GTACAGCCGGGAAGACAATAA | GCATTAAGGAGTCGGTTAGCA |
| **TNF-α** | GGTGCCTATGTCTCAGCCTC | CACTTGGTGGTTTGCTACGA |
| **TGF-β** | ATACGCCTGAGTGGCTGTCT | TCTCTGTGGAGCTGAAGCAA |
| **MyD88** | CACCTGTGTCTGGTCCATTG | AGGCTGAGTGCAAACTTGGT |
| **FOXp3** | TTCATGCATCAGCTCTCCAC | CTGGACACCCATTCCAGACT |
