# Supplementary material for: Early and late pulmonary effects of nebulized LPS in mice: An acute lung injury model
Source: PLoS One. 2017 Sep 27;12(9):e0185474. doi: 10.1371/journal.pone.0185474 (PMC5617199; doi:10.1371/journal.pone.0185474)
Supplement: S2 Table — (DOCX) [file pone.0185474.s003.docx]

**S2 Table. Immunohistochemistry standards.**

|  | **Antibody** | **Concentration** |
| --- | --- | --- |
| **Neutrophils** | Anti – MPO (Abcam – cod. ab9535) | 1:750 |
| **Lymphocytes** | Anti - CD3 (Serotec – cod. MCA1477) | 1:800 |
| **Macrophages** | Anti - MAC 2 (Cedarlane – cod. CL8942AP) | 1:100000 |
| **IL – 1ß** | Anti – IL-1ß (Santa Cruz – sc-7884) | 1:400 |
| **IL - 6** | Anti – IL-6 (Santa Cruz – sc-1265) | 1:1500 |
| **TNF - α** | Anti – TNF –α (Santa Cruz –sc-1348) | 1:3000 |
| **Collagen 1** | Anti – Col1a1 (Santa Cruz – sc-25974) | 1:100 |
| **MMP-2** | Anti – MMP2 (Santa Cruz – sc-8835) | 1:500 |
